# Challenges and recent advances in quantitative mass spectrometry‐based metabolomics

**DOI:** 10.1002/ansa.202400007

**Published:** 2024-06-26

**Authors:** Nathan Ghafari, Lekha Sleno

**Affiliations:** ^1^ Chemistry Department/CERMO‐FC University of Quebec in Montreal (UQAM) Montreal Canada

**Keywords:** internal standards, mass spectrometry, quantitative metabolomics, stable isotope labelling

## Abstract

The field of metabolomics has gained tremendous interest in recent years. Whether the goal is to discover biomarkers related to certain pathologies or to better understand the impact of a drug or contaminant, numerous studies have demonstrated how crucial it is to understand variations in metabolism. Detailed knowledge of metabolic variabilities can lead to more effective treatments, as well as faster or less invasive diagnostics. Exploratory approaches are often employed in metabolomics, using relative quantitation to look at perturbations between groups of samples. Most metabolomics studies have been based on metabolite profiling using relative quantitation, with very few studies using an approach for absolute quantitation. Using accurate quantitation facilitates the comparison between different studies, as well as enabling longitudinal studies. In this review, we discuss the most widely used techniques for quantitative metabolomics using mass spectrometry (MS). Various aspects will be addressed, such as the use of external and/or internal standards, derivatization techniques, in vivo isotopic labelling, or quantitative MS imaging. The principles, as well as the associated limitations and challenges, will be described for each approach.

**Abbreviations**
CIL: chemical isotope labelling
DESI: desorption electrospray ionization
IS: internal standard
LC: liquid chromatography
MALDI: matrix‐assisted laser desorption ionisation
MRM: multiple reaction monitoring
MS: mass spectrometry
MS/MS: tandem mass spectrometry
MSI: mass spectrometry imaging
qMSI: quantitative mass spectrometry imaging
SCFA: short‐chain fatty acids
TIF: tumor interstitial fluid

## 1 INTRODUCTION

### 1.1 Metabolomics

Metabolomics aims to study the variation of small biological molecules, or metabolites, often to better understand perturbations involved in disease or environmental stress. These metabolites play major roles in biochemical reactions within cells. Detecting and quantifying these compounds can therefore provide useful information on the physiological state of an organism. When an organism is subjected to an internal or external stimulus, metabolic variations often occur before the first clinical signs.^1^, ^2^ Detecting and quantifying the variations at the metabolome level associated with a specific perturbation is an important step for improving our understanding of the biological mechanisms involved and can also serve to assess the impact of a specific therapy at the molecular level. Metabolomics can be applied to many different areas such as biomarker discovery, physiology, nutrition, microbiome analysis, exposomics, and environmental assessment.^3^

Over the past ten years, less than 8% of metabolomics studies have implicated quantitative metabolomics (based on a Pubmed search using keywords “metabolomics” and “quantitative metabolomics” accessed on 12 Feb 2024). However, quantitative metabolomics is steadily increasing, with the number of papers doubling over the past 8 years, to over 800 publications in 2023. Quantitative metabolomics has many analytical challenges, one of them is the nature of the analytes themselves. Metabolites are a very broad category of compounds, defined loosely as endogenous molecules with molecular weights less than 1500 Da, but with a wide range of physico‐chemical properties.^4^ Some metabolites are very small and highly polar, like amino acids or organic acids, while others are much less polar, such as lipids. Powerful analytical techniques, such as liquid (or gas) chromatography coupled with mass spectrometry (LC‐MS or GC‐MS) or nuclear magnetic resonance, are required for proper characterization and precise quantitation of metabolites in these complex biological samples. These platforms are essential tools for providing qualitative and quantitative insights into metabolomics studies. Due to its sensitivity and versatility, LC‐MS is an excellent technique for metabolomics. It enables the separation, detection, and quantitation of a wide range of metabolites, even at very low concentrations. In the case of the analysis of samples in complex matrices, the use of separative techniques, such as liquid chromatography, is primordial. These techniques help to remove potential interferences, and different approaches can be taken for the analysis by mass spectrometry. Most studies involving absolute quantitation are carried out using targeted assays, most often triple quadrupoles in multiple reaction mode^5^, ^6^, ^7^, ^8^ where accurate and robust quantitation can be achieved by choosing selective transitions with optimized instrumental parameters. Absolute quantitation can also be performed through less targeted analyses, using high‐resolution mass spectrometry,^9^, ^10^, ^11^, ^12^ such as quadrupole‐time‐of‐flight or orbitrap‐based systems. In these cases, data are acquired in an untargeted manner, and selective data processing allows peaks of interest to be filtered out for further analysis. This review focuses on different workflows for quantitative metabolomics using mass spectrometry while highlighting recent advances in this field.

### 1.2 Untargeted & targeted metabolomics

In most metabolomics studies, one of two approaches can be chosen, the untargeted approach (also known as metabolic profiling) and the targeted approach (often referred to as quantitative metabolomics). The untargeted approach will include a step for the identification of detected metabolites, followed by their relative quantitation, most of the time by comparing multiple groups within the same dataset. This approach can be useful as a hypothesis generation strategy.^13^ The targeted approach is more of a hypothesis‐testing strategy, as it focuses on quantifying specific, predetermined metabolites. Usually, the number of studied metabolites is much smaller in a targeted assay. As a result, the amount of data obtained is significantly different from the non‐targeted approach. The targeted approach is usually highly sensitive and selective, making it more suitable for absolute quantitation.

### 1.3 Quantitative metabolomics

Having access to the absolute concentration of metabolites represents the gold standard for quantitative studies since results are more reliable and reproducible than the relative comparison between two different groups. In the latter scenario, combining data from different studies is essentially impossible, since the observed variations are based on signal intensities which can fluctuate significantly between batches even using the same sample preparation and analysis method. Absolute quantitation overcomes this limitation and enables the comparison between studies, however, determining endogenous metabolite concentrations in complex biological matrices involves several analytical challenges.^13^

Biological variability between samples, as well as analytical variability, must be considered and corrected.^14^, ^15^ Some biological variability can be addressed via pre‐acquisition normalization, while analytical variations are corrected with post‐acquisition normalization techniques prior to data processing. Many studies have described different strategies for this.^16^, ^17^, ^18^, ^19^, ^20^, ^21^ The extraction of metabolites of interest can also be optimized for a targeted workflow. Many types of extraction protocols for specific metabolite classes in different types of samples have been described.^22^, ^23^, ^24^

Method optimization in quantitative metabolomics is crucial for enhancing the sensitivity, accuracy, and reproducibility of metabolite detection. Optimizing the separation of metabolites^25^ from complex samples, for instance, will help reduce matrix effects due to the co‐elution of many species. The quantitation of endogenous metabolites is particularly difficult, as they are present in the background biological matrix. Absolute quantitation of these analytes requires techniques to ensure the reliability of quantitative metabolomic analyses. In this review of quantitative metabolomics, we first introduce some basic concepts and important caveats to quantifying endogenous metabolites in complex biological samples, while highlighting different calibration approaches. Four different approaches will be covered, including stable isotope dilution, in vivo labelling, chemical isotope labelling, and quantitative mass spectrometry imaging.

### 1.4 Method validation and quality control in metabolomics

When quantifying metabolites in complex matrices, certain method validation steps are needed to ensure the quality and accuracy of the results. Validation of an analytical method typically involves a series of experiments following established protocols and guidelines to assess the method's performance. Many parameters can be determined during the method validation process, such as reproducibility, linearity, or associated uncertainties associated with the results. Several tools are used to measure these parameters, such as the use of certified reference materials, containing analytes in known concentrations and can be used to ensure accuracy and precision. An essential step in metabolomics method validation is the use of interlaboratory comparisons. This not only confirms the method's performance but also allows the precision and accuracy of the results to be determined with greater confidence. In recent years, with the increasing development of metabolomics, method validation and quality assessment have become a key criterion in these studies. For further information, the readers are referred to more detailed studies focusing on these aspects.^26^, ^27^, ^28^, ^29^, ^30^, ^31^, ^32^

## 2 ANALYTICAL STANDARDS FOR ABSOLUTE QUANTITATION

### 2.1 External calibration curves in artificial or depleted matrices

One of the most common and robust techniques for the absolute quantitation of target compounds is based on external calibration curves.^33^, ^34^ However, when the analytes are present endogenously in the background matrix, this technique is not as straightforward as for quantifying drugs or other xenobiotics, which is done routinely for pharmacokinetics and bioavailability studies with plasma and urine samples. The simplest solution is spiking the analytes, at different concentrations to produce an external calibration curve without a background matrix. However, this strategy does not consider important matrix effects, including differences in sample recovery, as well as ion suppression or enhancement during LC‐MS analysis due to other components in the sample that co‐elute with the analytes of interest. Therefore, the chemical environment of the calibration curve should be as similar as possible to the samples studied to correct for these effects. To confirm that an external calibration curve in a simplified matrix is suitable for the quantitation of an endogenous metabolite, a parallelism assay can be performed by comparing a sample‐dilution response curve to the standard curve.^35^ If the slopes are comparable between the diluted sample response curve and the external calibration curve, the assay is deemed acceptable for quantitation.

**FIGURE 1:**
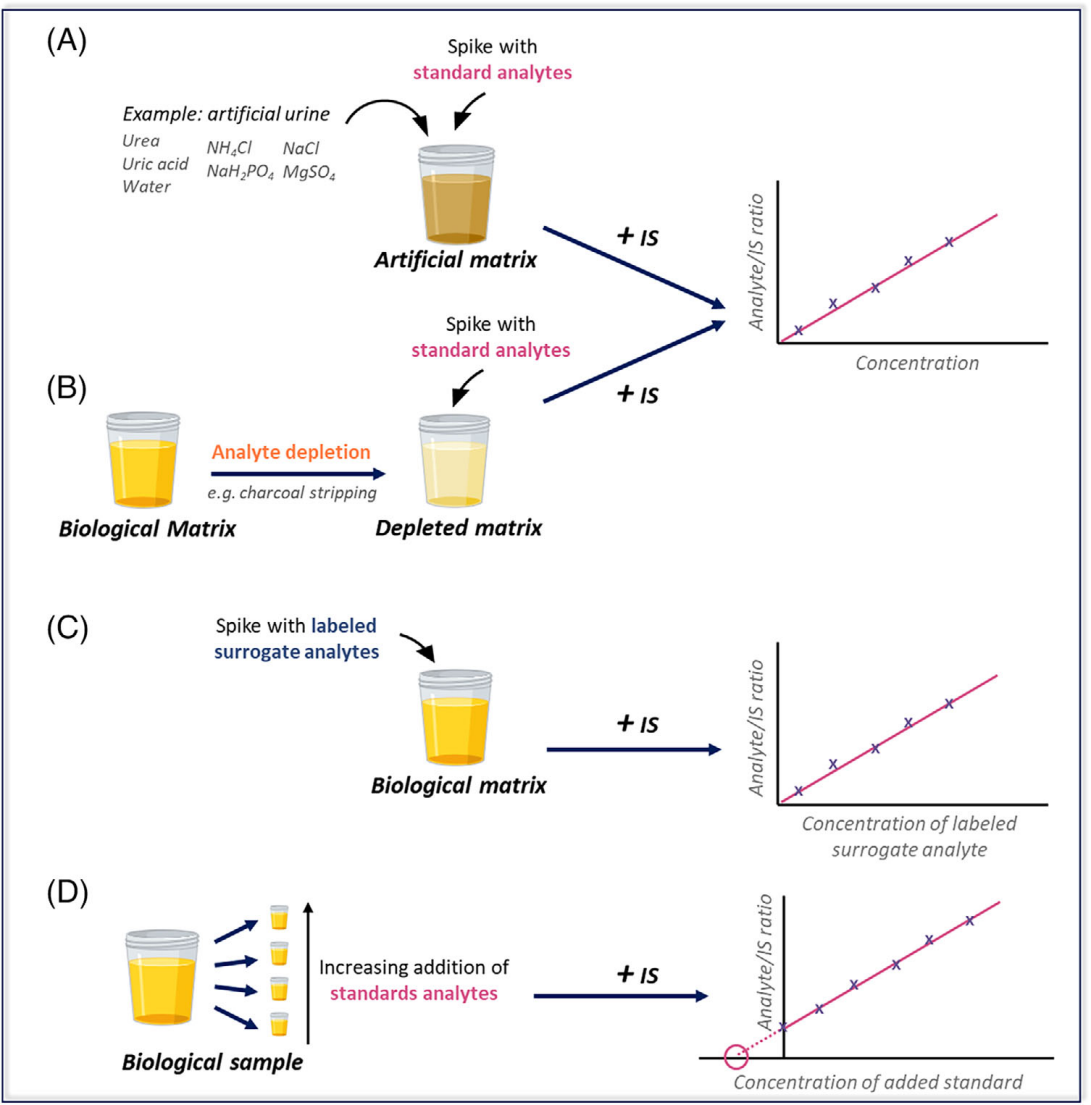
General workflow for absolute quantitation using external calibration with internal standard (IS) in an artificial matrix (A), analyte‐depleted matrix (B), using labelled surrogate analytes in the authentic matrix (C) or by standard addition with increasing concentration of analytes added to each study sample (D).

To help solve these limitations, several methods have been developed, one of which is to create an artificial matrix containing the major compounds found in the biological matrix, but without the analytes of interest.^36^, ^37^, ^38^ This artificial matrix should contain the principal components of the real sample, including those responsible for ion suppression, for instance. This approach has become standard practice in certain types of studies, such as urine analysis.^39^, ^40^, ^41^ The use of artificial urine is relatively common, and protocols exist to prepare this artificial matrix from a mixture of salts, creatinine, urea, and uric acid^42^, ^43^ (Figure 1A). However, this approach requires many preparation steps, and the replicated matrix can still be quite different from the real samples. Plus, some matrices are extremely complex and therefore very hard to imitate, such as plasma or tissue homogenates.^44^ Thus, this approach, although very useful, is not practical for most studies involving complex biological matrices.

In the case where the analyte is not naturally present in the biological matrix, the most straightforward approach is to directly spike the standards in a control (analyte‐free) matrix and use it to quantify the real sample. One solution for obtaining complex biological matrices that do not contain the analyte of interest is to remove the target compounds from the biological matrix and use the resulting depleted matrix to construct a traditional external calibration curve^45^, ^46^, ^47^, ^48^ (Figure 1B).

Many protocols have been published using activated charcoal, for example, to remove metabolites in plasma or urine. A recent example has used this strategy for the quantitation of isoprenoids.^49^ Some depleted matrices have been commonly used and even commercialized, such as charcoal‐stripped serum (depleted of hormones^50^) or silica‐treated serum (lipid‐depleted^51^). Although very useful, these approaches still have limitations. Matrix effects are particularly difficult to correct when using artificial matrices,^52^, ^53^ and not all studies lend themselves to depleted matrices. Through this depletion step, certain compounds responsible for important matrix effects can be removed, yielding a similar problem as an artificial or solvent matrix. To properly correct for matrix effects, losses during sample preparation or variable instrumental sensitivity, internal standards are usually added to the calibration standards and samples. A better understanding of the challenges associated with a given matrix type would help design more complex and accurate surrogate matrices for absolute quantitation.

**FIGURE 2:**
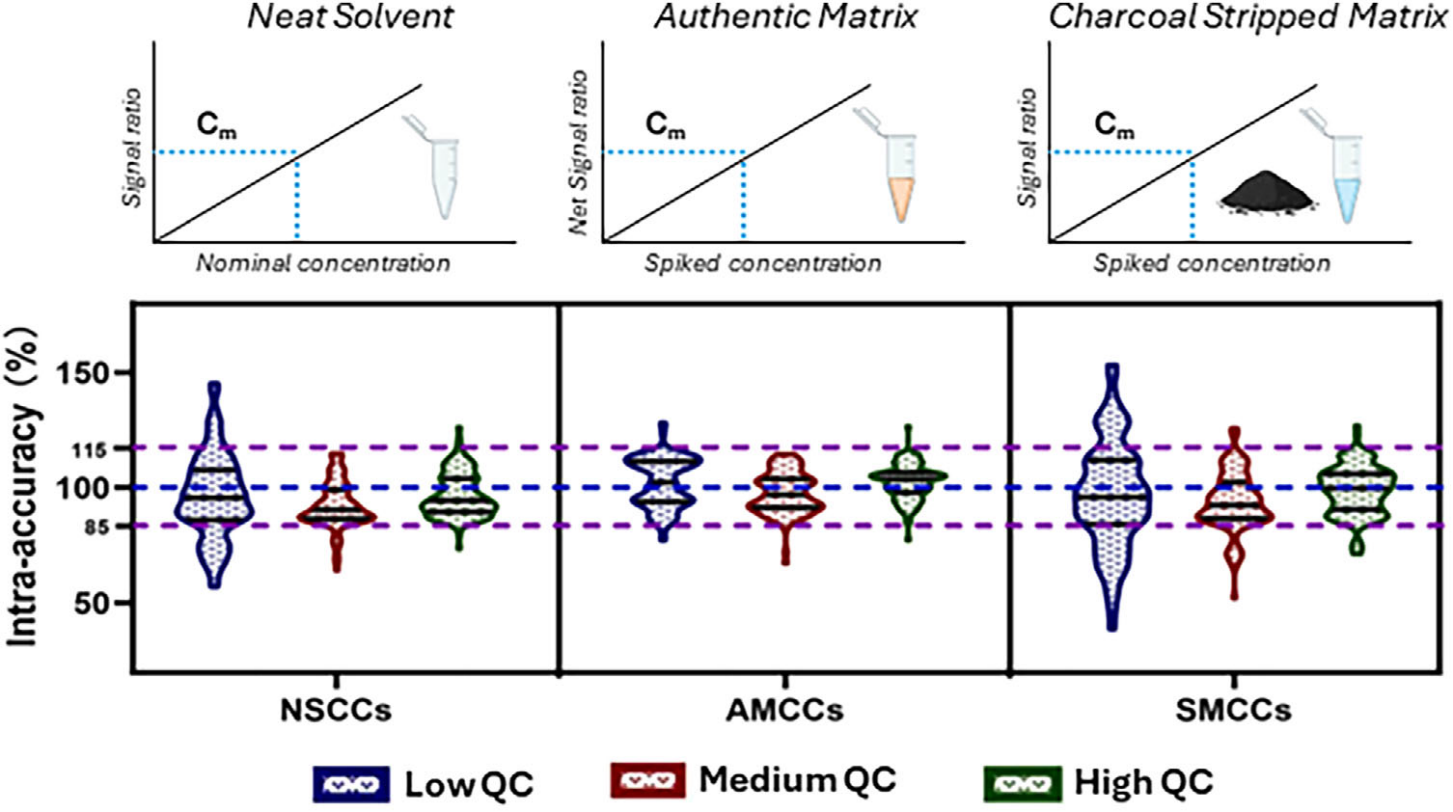
Three different calibration methods were used for the quantitation of bile acids in rat serum, in a neat solvent, authentic matrix and charcoal‐stripped serum. Violin plots of the accuracies were obtained using a pooled rat serum spiked with three concentration levels of spiked analytes. Figure adapted from Yang et al.^54^

A recent study by Yang et al.^54^ compared different calibration methods for the quantitation of bile acids in rat serum, including calibration curves with labelled internal standards in a neat solvent, in the authentic matrix and in the charcoal‐stripped matrix (Figure 2). The three methods were compared using a pooled rat serum sample, spiked with known amounts of bile acids at three levels, and background signals from endogenous levels in the authentic matrix were subtracted from the measured signals (referred to as net signal in the figure) to construct the calibration curve. Their results showed that the calibration in an authentic matrix yielded the best accuracy.

Accurate and robust quantitation by LC‐MS requires the use of an appropriate internal standard (IS), having similar properties to the analyte and, above all, not naturally present in the samples. Isotopically labelled internal standards have thus become the gold standard. Isotopically labelled molecules share chemical characteristics, similar extraction efficiency during sample preparation, co‐eluting chromatographic retention times, and similar response factors as their corresponding unlabelled versions, but with an appropriate mass shift to differentiate them by mass spectrometry.

An isotopically labelled standard (or mix) can be added directly into the sample matrix (usually from pooled samples or commercially available matrix) to serve as a surrogate analyte at increasing concentrations and the labelled calibration curve can be used for quantitation, correcting matrix effects, efficiency and recovery (Figure 1C). Another approach commonly used is to spike the labelled standard directly in the sample, ideally at a concentration relatively close to the analyte to perform a one‐point calibration to determine the concentration.^55^, ^56^ It is also important to verify that for a chosen method the stable isotope labelled to be used as a surrogate analyte would yield identical response factors as the unlabelled metabolite. In general, this should be the case, if labelled atoms do not interfere with properties affecting ionization, or fragmentation in the case where quantitation is done via MS/MS (for example, with multiple reaction monitoring [MRM] analysis).

### 2.2 Standard addition method

Another method that can be applied to quantify endogenous metabolites is standard addition (Figure 1D). A reference standard (or a mix of standards) is added at increasing concentrations to several aliquots of the same sample. Using the curve obtained from the resulting samples allows the concentration of the initial sample to be determined. In this way, quantitation can be achieved while compensating for matrix perturbations.^7^ However, this approach is rarely used in metabolomics studies, for several reasons. Firstly, the standard addition method requires a large volume of each sample, which can be difficult to access when analyzing biological samples. Another limiting factor is that this technique can achieve the most accurate quantitation if the spiked standards are in a concentration range close to that of the analyte, and therefore requires prior knowledge. This technique is also much slower as several injections are needed to quantify each sample, usually at least five times the analysis (and preparation) time for a proper calibration to be achieved.

## 3 IN VIVO STABLE ISOTOPE‐LABELLED STANDARDS

### 3.1 Generation of labelled standards in vivo

For accurate absolute quantitation in complex biological matrices, the gold standard is to use an isotopically labelled internal standard for each analyte, where deuterated and ^13^C‐labelled analogues are widely employed. However, despite their many qualities, isotope standards still present important constraints. One major drawback is their substantial cost and lack of availability for many metabolites. Quantifying a long list of metabolites requires access to numerous standards, therefore, most studies are still limited to reporting relative quantitation results.

**FIGURE 3:**
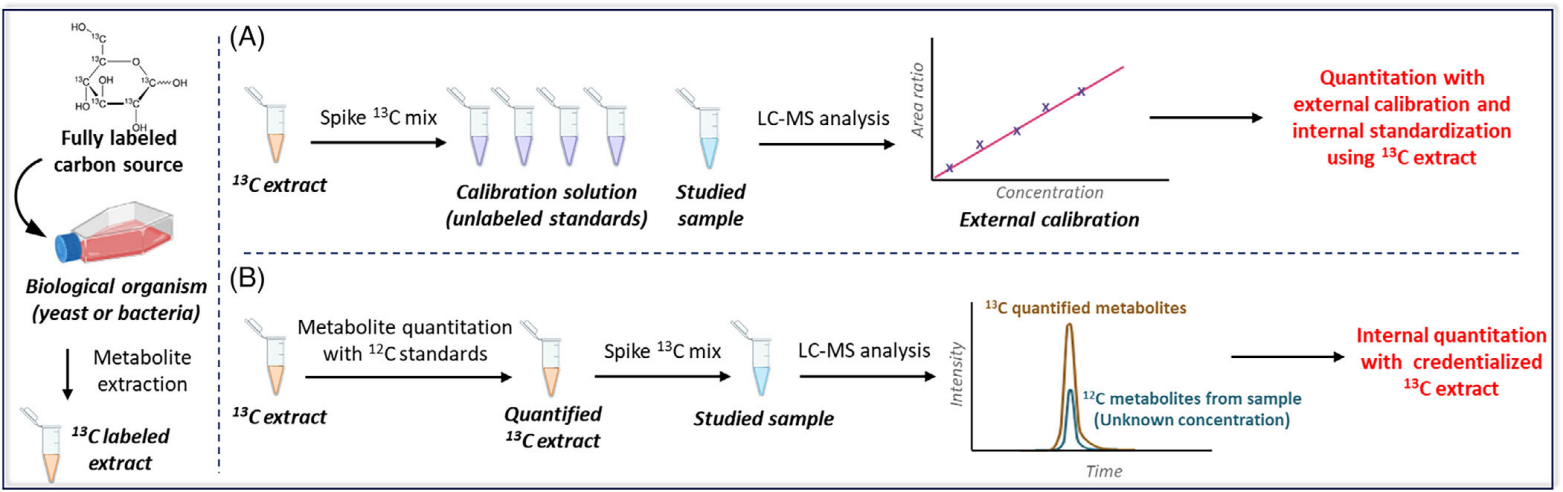
General workflow of using isotopically labelled standards generated in vivo for absolute quantitation, either by using labelled metabolite extract as an internal standard (IS) mix (A) or via internal calibration using a credentialized extract (B).

Recently, approaches have emerged to provide more cost‐effective access to labelled standards. The most widely used method is to grow biological organisms in labelled media, known as in vivo labelling. For example, bacterial cultures can easily grow using fully ^13^C‐labelled glucose as their sole carbon source,^57^, ^58^, ^59^, ^60^, ^61^ resulting in the production of labelled metabolites. This approach makes it possible to obtain many isotopically labelled metabolites, at a fraction of the cost of commercial standards. The only isotope‐labelled compounds needed are the precursors used for the biosynthesis of metabolites. These in vivo metabolites can then be used as internal standards for absolute quantitation. Two approaches are frequently used, the most common one creating external calibration curves using unlabelled standards. Then, the ^13^C metabolite extract is added to the calibration solution and to the study samples, with the extract acting as an internal standard mix of labelled metabolites. It is then possible to quantify the metabolites in the sample using external calibration with internal standardization, and if each analyte of interest has its corresponding isotopically labelled version for normalization purposes, the differences in matrix effects, when considering the peak area ratios (analyte/isotopically‐labelled metabolite), between the calibration curve and the study samples should be minimized (Figure 3A). The second approach requires an initial step to quantify the labelled metabolites in the produced extract. As the entire extract is isotopically labelled, it is possible to quantify these metabolites using unlabelled compounds, resulting in a credentialized metabolite extract. Several approaches can be used to quantify labelled metabolites in the extract. The most straightforward is to create an external calibration curve using unlabelled metabolite standards and perform an external quantitation. If the extract is uniformly labelled, it is also possible to spike directly unlabelled standard into it, as a surrogate analyte, and perform internal quantitation. A recent study by Zhong et al.^62^ has used this approach to quantify ^13^C metabolites obtained from *Escherichia coli*. By spiking unlabelled metabolite standards directly into the ^13^C extract, they were able to quantify the labelled metabolites in *E. Coli*. Once quantified, the extract can then be directly spiked into all samples and used for a one‐point calibration (Figure 3B). It is important to note that, in this case, accurate quantitation is best achieved if the concentrations in the ^13^C mix are close to those of analytes of interest in the samples. One possible solution is to spike the credentialized labelled extract at different ratios within each sample, to create a broader calibration curve.

The biosynthesis of isotopically labelled metabolites in vivo, however, remains limited by several aspects. Firstly, the pool of metabolites generated is limited to the naturally occurring metabolites present in the organism used for the labelling experiment. Some metabolites are unique to certain biological matrices or organisms and will therefore not be naturally present in the ^13^C‐labelled bacterial extracts, for instance. Currently, there exist a few established protocols for isotopically labelling metabolites in bacteria (*E. coli*) or yeast (*Saccharomyces cerevisiae* and *Pichia pastoris*), where the ability to absolutely quantify compounds in study samples would be limited to those present in the labelled organisms.

**FIGURE 4:**
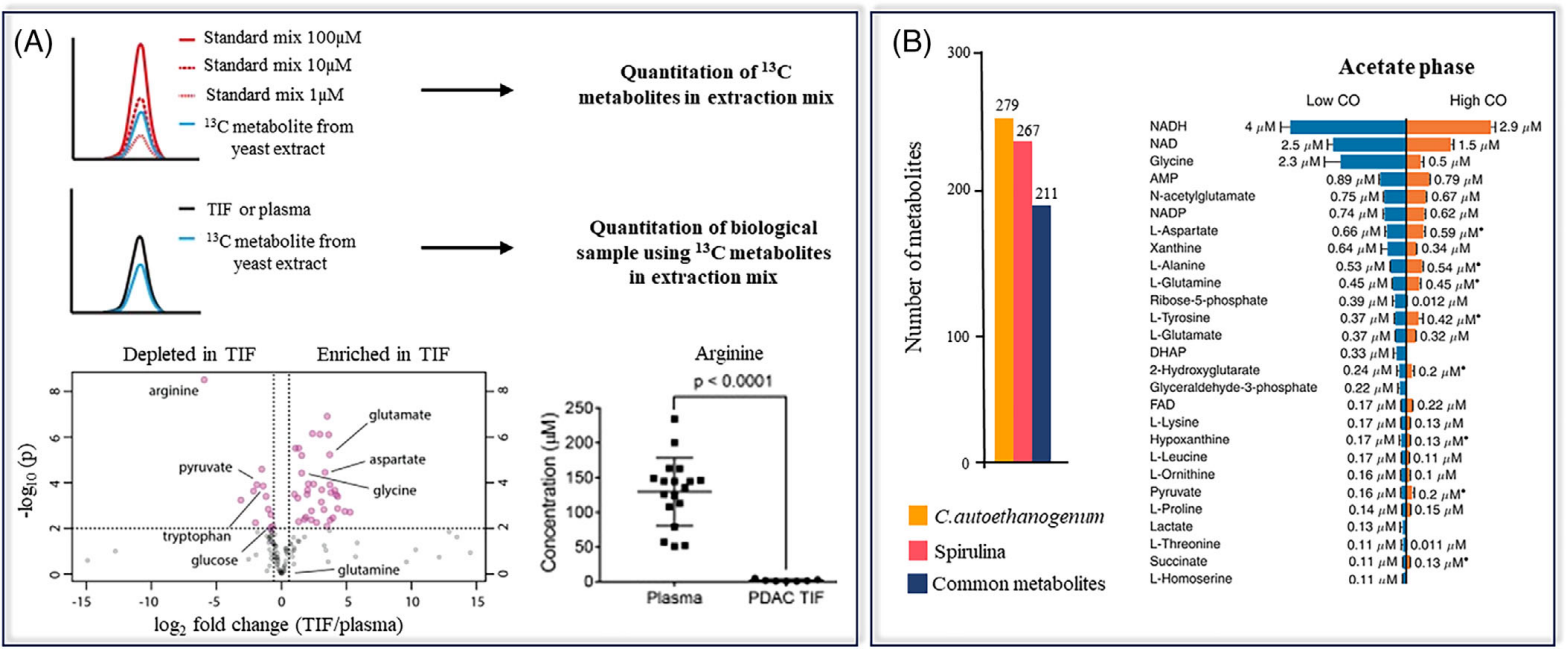
(A) Workflow for quantifying a ^13^C labelled extract before adding in tumour interstitial fluid (TIF) and plasma samples for internal (one‐point) calibration. Volcano plot shows the variation of metabolites between plasma and TIF and an example of absolute concentrations measured for arginine using this method. Figure adapted from Sullivan et al.^63^ (B) Bar plot representing the metabolites overlap between spirulina and Clostridium autoethanogenum, and resulting Clostridium autoethanogenum metabolites levels found under low and high CO conditions using ^13^C labelled and quantified spirulina extract, adapted from Schatschneider et al.^64^

In some studies, the organisms used as a source for labelled metabolites are different from the actual samples. Sullivan et al.^63^ (Figure 4A) have used this approach for quantifying metabolites in mouse models of cancer. They first quantified metabolites in a ^13^C yeast extract using an external calibration approach with unlabelled standards at different concentrations. The labelled extract was then used as an internal standard mix to determine the absolute concentration in plasma and tumour interstitial fluid (TIF) samples, where 70 metabolites were quantified. In another study, Schatschneider et al.^64^ (Figure 4B) investigated the use of fully labelled spirulina as a source of internal standards for simultaneous quantitation of 74 metabolites from Clostridium autoethanogenum.

Although very useful, most in vivo labelling remains limited to relatively simple organisms, such as yeast^65^, ^66^, ^67^, ^68^ and bacteria.^62^, ^69^, ^70^ Some studies have proposed protocols to generate isotopically labelled different species such as *C. elegans* ^71^, ^72^ or mice,^73^ with more advanced labelling techniques. Continued advancement in this field will contribute to the development of quantitative metabolomics in complex organisms.

In vivo labelling can also be used in other applications, such as in the field of fluxomics, which employs specifically labelled precursors for studying metabolic reactions, and the regulation of certain pathways. These studies are highly informative to specifically interrogate the dynamics in metabolic variations. For more in‐depth information, readers are encouraged to explore recent studies focusing specifically on this approach.^74^, ^75^, ^76^, ^77^, ^78^

### 3.2 Commercial kits for absolute quantitation

Absolute quantitation of metabolites presents various challenges, such as the need for appropriate standards, including labelled internal standards, as well as optimized and targeted methods. Some of these challenges require significant financial resources or knowledge of specific fields making quantitative metabolomics analysis less accessible. To address these issues, several companies and organizations have been proposing kits for targeted and quantitative metabolomics analyses. These kits enable quantitative studies to be carried out on numerous compounds from several categories by offering a ready‐to‐use workflow, with chromatographic methods and MS parameters optimized for each class of compound and for different mass instruments. By using different approaches, such as derivatization (see section below) or direct injection without chromatography for specific compounds, this type of kit can be used for certain metabolite categories that usually require an optimization step or multiple methods. In certain cases, the broad range of metabolites covered enables a more exploratory approach, while maintaining a quantitative dimension. Many recent studies have used such kits to investigate metabolic variations in complex samples, such as urine^79^ and faeces.^80^ Using a commercial kit‐based assay, Franco et al.^81^ studied lipid dysregulation caused by exposure to environmental contaminants by quantifying ceramides, triglycerides, and phosphatidylcholines in human liver cells. In another study, Pedrafita et al.,^82^ tryptophan and kynurenine metabolites were quantified in mouse urine to study metabolic perturbations caused by acute kidney injuries.

## 4 CHEMICAL ISOTOPE LABELLING

Due to the broad structural diversity of metabolites, different methods need to be applied to cover a wide range of metabolites. Very polar metabolites, for instance, are difficult to separate by traditional reverse‐phase chromatography, and the detection of minor compounds can also be suppressed by coeluting compounds. Background signals can also often have a deleterious impact on the detection of species with very low molecular weights. These issues often require the combination of multiple techniques, decreasing the overall analysis throughput.^83^ Many techniques have been developed to address this problem, including the use of chemical derivatization to improve the detectability of some more problematic substances and enable their analysis using more generic workflows. Some compounds lend themselves particularly well to derivatization, and this technique is now common for the analysis of amino acids,^84^, ^85^, ^86^ steroids^87^, ^88^ and short‐chain fatty acids (SCFA),^89^, ^90^, ^91^ among others. In addition to these advantages, incorporating isotope labels through the derivatization process provides access to a quantitative dimension.

### 4.1 Quantitation using derivatization with labelled reagents

**FIGURE 5:**
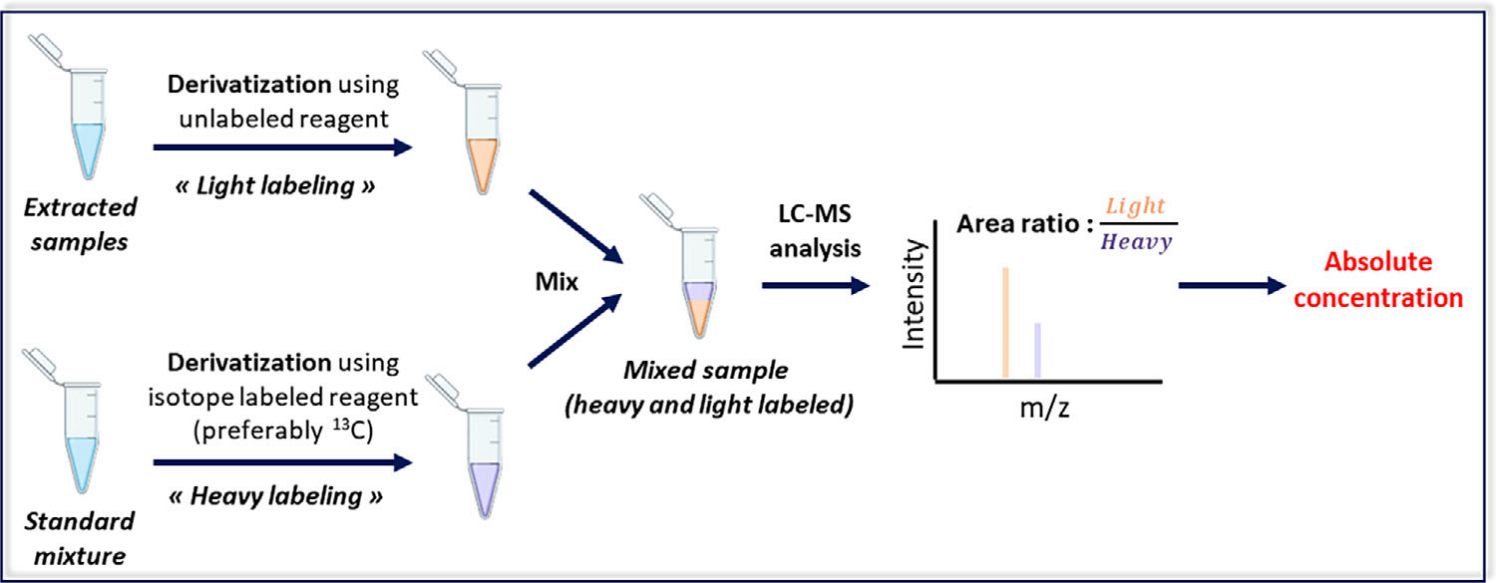
General workflow for absolute quantitation via chemical isotope labelling.

Quantitative chemical isotope labelling (CIL) is usually based on the derivatization of biological samples using an unlabelled reagent and a mixture of analytical standards with an isotopically labelled reagent. These two derivatized samples are then mixed prior to analysis. The peak area ratios (unlabelled/labelled) for derivatized metabolites can be used to determine their concentrations (Figure 5). Some studies use a similar methodology with pooled samples. The pooled sample is first quantified as described above and then used as a labelled sample to quantify the other samples.^92^ An alternative approach exists where the derivatization reagents are not isotopically labelled, but the standard mixture is.

**FIGURE 6:**
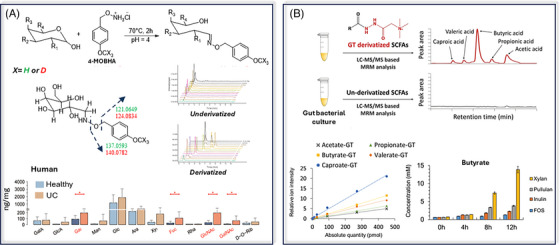
(A) Derivatization reaction between monosaccharides and *O*‐(4‐(methoxy)benzyl)‐hydroxylamine (MOBHA). A comparison of chromatographic separation is shown with and without derivatization, as well as fragmentation patterns for labelled and unlabelled derivatized compounds. The bar plot represents determined concentrations in healthy and ulcerative colitis faecal samples, adapted from Wang et al.^95^ (B) General workflow used for the quantitation of short‐chain fatty acids in gut microbiota using Girard's reagent T (GT), with increased detectability, with external calibration curve used for the quantitation of five short‐chain fatty acids (SCFAs) shown. The bar plot represents butyrate concentrations at different times when using five different plant‐derived carbohydrate sources, adapted from Song et al.^89^

Many studies have used CIL to quantify metabolites in complex biological matrices. Liu et al.^88^ used this approach to quantify steroids in rat urine with LC‐MRM. Two different reagents, 4‐dimethylamino‐benzoic acid and Girard's reagent P were used for derivatization, with their deuterated analogues for quantitation. In another study, Achaintre et al. quantified 37 polyphenols, using unlabelled and labelled versions of dansyl‐chloride, in urine^93^ and plasma.^94^ A CIL approach was also applied^95^ (Figure 6A) to quantify monosaccharides in faecal samples. Using methoxy‐benzyl‐hydroxylamine for derivatization, improved chromatographic separation and sensitivity of 12 monosaccharides in human and mouse faeces enabled perturbations in ulcerative colitis (UC) to be studied. A similar approach was used by Song et al.^89^ to quantify SCFA in gut microbiota (Figure 6B). To create an external calibration curve, a mixture of SCFA standards was derivatized with Girard's reagent T and deuterated butyrate was used as the internal standard. Various SCFAs were quantified in E. rectale extracts to study the impact of different carbohydrate sources.

### 4.2 Advantages of derivatization

In addition to providing accurate quantitation, CIL using derivatization can often improve the sensitivity of metabolites, usually by increasing their hydrophobic character and facilitating separation on reverse‐phase columns.^83^, ^96^, ^97^, ^98^ The use of reagents possessing a characteristic group, such as bromine or quaternary amine group, can facilitate the detection of certain compounds.^99^, ^100^, ^101^, ^102^ Another advantage is the possibility of using other detection techniques to quantify the total amount of derivatized metabolites for normalization purposes.^103^, ^104^, ^105^ Some derivatization agents, such as dansyl or phenylisothiocyanate, have unique UV absorbance or fluorescence properties amenable to normalization.

### 4.3 Challenges and limitations of quantitative CIL

Although very useful, CIL also presents a variety of challenges. The choice of reagent must be adapted to the type of sample and compounds being targeted. Each reagent will also derivatize a specific subset of molecules, for example, dansyl chloride^106^ reacts with metabolites with amines and phenols. The chemical selectivity of these reagents, while very useful for targeted studies, is a limiting factor when it comes to studying metabolites from different chemical classes.^107^ It is then necessary to use several reagents, requiring longer and more complex sample preparation. Li et al.^105^ have described a four‐channel chemical isotope labelling approach applied for quantitative metabolome analysis in different food products.

Derivatization reaction conditions are also an important limiting factor. Many metabolites are unstable or easily degraded,^108^, ^109^, ^110^ therefore conditions involving high temperature and extreme pH can lead to the degradation of certain metabolites. The addition of reagents and solvents often also dilutes the sample which can necessitate an extra concentration step during sample preparation.

The choice of isotope label is also important to consider. For instance, when deuterated compounds are used, the isotopically labelled versions usually elute slightly earlier than non‐deuterated ones, which can affect metabolite pair filtering and accurate quantitation. For this reason, a ^13^C‐labelled reagent is therefore preferred, since these labelled are much less susceptible to retention time variation.

Also, derivatization influences the potential for identification via tandem mass spectrometry.  In many cases, there are only very minor fragments, stemming from specific fragmentation of the original molecule,^111^ making identification of unmodified metabolites very difficult. The creation of spectral databases of derivatized metabolites^112^, ^113^, ^114^ or the application of computerized prediction tools^112^, ^115^ is being implemented as a solution to this problem.

## 5 QUANTITATIVE MASS SPECTROMETRY IMAGING

### 5.1 Challenges for mass spectrometry imaging metabolomics

Spatially resolved metabolomics is a powerful tool to monitor the distribution of specific compounds within a tissue.^116^, ^117^, ^118^ Accurate quantitation of metabolites in different tissue regions, for instance, can provide critical information about the progression of a disease and the treatment efficacity. Several papers have described the potential and analytical aspects of mass spectrometry imaging (MSI) in metabolomic studies.^119^, ^120^, ^121^, ^122^, ^123^

However, matrix effects can limit the ability to quantify metabolites in MSI.^124^, ^125^ In traditional LC‐MS workflows, these effects are decreased via sample purification and chromatographic separation. Strategies to lessen variability in quantitative (qMSI) include automated matrix‐assisted laser desorption ionisation (MALDI) matrix application, internal standard addition, signal calibration or data normalization.^126^ An important limitation is the heterogeneity of a tissue sample, causing potentially very different ionization responses, and background signals, between regions. During the past few years, many studies have explored and optimized different techniques to increase reproducibility and the ability for accurate absolute quantitation. Here, we provide an overview of different strategies used for qMSI in metabolomics. For additional information, there are several reviews focusing on qMSI.^127^, ^128^, ^129^, ^130^, ^131^, ^132^

#### 5.1.1 MALDI qMSI for metabolomics

**FIGURE 7:**
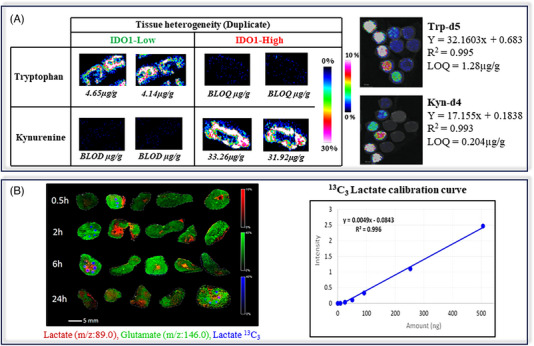
(A) Spatial resolution of tryptophan and kynurenine levels in tumour tissues using matrix‐assisted laser desorption ionization mass spectrometry imaging (MALDI MSI), with spots of labelled standards for calibration. Adapted from Ait‐Belkacem et al.^135^ (B) Distribution of lactate and glutamate levels in mouse tumours over 24 h. Calibration curve used for lactate quantitation was obtained via a ^13^C‐labelled standard. Adapted from Swales et al.^136^

Even though MALDI is mostly used for studying large biomolecules, such as proteins or biopolymers, several research groups have investigated MALDI for metabolomics studies as a powerful tool for imaging applications. Due to the limitations of a solid biological sample, compensating for matrix effects is crucial for quantitation.^127^, ^133^, ^134^ The simplest solution is to create a calibration curve in solution spotted on the MALDI target, using an internal standard spotted both on tissue samples and in calibration standards for normalization, however, this approach does not correct for effects of the biological matrix on ionization response. Alternatively, isotope‐labelled standards can be directly spotted on the tissue sample to create a calibration curve helping correct for matrix effects. For example, Ait‐Belkacem et al.^135^ (Figure 7A) implemented MALDI‐qMSI to monitor the metabolic response in murine tumour models, with different enzyme levels of indoleamine‐2,3‐dioxygenase 1 (IDO1), by quantifying tryptophan and kynurenine. Using on‐tissue spotting of deuterated standards, a calibration curve was obtained for each analyte. In a similar study, Swales et al.^136^ (Figure 7B) quantified the distribution of lactate and glutamate in a mouse tumour model, using labelled standards to create a calibration curve. Their results showed that glutamate was evenly distributed, but lactate was heterogeneously localized in the (pre)necrotic regions.

In addition, the presence of the organic MALDI matrix results in significant background signals in the low mass area, making the detection of certain compounds very difficult. Heterogenous matrix crystal formation during the spraying process can also cause spot‐to‐spot variability.^133^ These limitations, although partially amendable, have prompted some research groups to explore other imaging approaches that do not require the use of a matrix.

### 5.2 Desorption electrospray ionization imaging for quantitative metabolomics

Desorption electrospray ionization (DESI) requires minimal sample pretreatment, without the need for an added matrix for ionization, making it a preferred technique for MSI of small metabolites. In a recent study, Gerbig et al.^137^ performed DESI‐MS to analyze pesticides in different fruits. Using an external calibration curve and labelled internal standards for normalization, they were able to quantify pesticides at low concentrations (< 10 mg/kg). They compared their results with a reference LC‐MS/MS method, showing that the values obtained were in the same range.

However, the spatial resolution offered by this technique is limited compared to MALDI due to the absence of a focused laser. To enhance resolution, several variants are being developed, such as nano‐DESI,^138^ with its improved spatial resolution and sensitivity showing promise for quantitative spatial metabolomics even in single cells. Bergman et al.^139^ were able to detect amino acids and phospholipids in human cheek cells using nano‐DESI. With a phosphatidylcholine (PC) standard directly added to the DESI solvent, they quantified seven PCs in a single cell. Marques et al.^140^ used a pneumatically assisted nano‐DESI to observe the distribution of several metabolites and lipids in cells. Interestingly, a heterogeneous distribution of metabolites was seen, with a more uniform distribution of lipids.

Although there are still relatively few studies involving quantitative metabolomics using DESI, and most of them focus on lipids, recent publications suggest that this technique has many advantages, including the potential for adding standards directly to the source solvent for absolute quantitation. There remains the issue of ion suppression, therefore less abundant or poorly ionizable metabolites are likely not amenable to quantitation using this technique. The development of additional techniques like airflow‐assisted DESI^141^ is also showing promise for expanding the range of metabolites detectable in MSI, facilitating its use in complex metabolomic studies.

## 6 SUMMARY AND OUTLOOK

This review has described the principles and challenges of quantitation techniques used in metabolomics in recent years. It is important to note that there is no gold standard in quantitative metabolomics. The choice of technique used must be guided by several criteria, such as the type of sample studied, the analytes to be quantified and the instrumentation available. Several examples of quantitative metabolomics studies using the different approaches described are listed in Table 1.

**TABLE 1:** Representative examples of quantitative metabolomics studies

Method used	Techniques	Targeted compounds	Aim of the study	Ref
**Analytical standards for absolute quantitation**	External calibration in charcoal‐stripped plasma	Indole	Monitor levels of indole in mouse serum and tissue	45
	External calibration in charcoal‐stripped plasma	Isoprenoid	Method for quantifying isoprenoids in plasma and cells	49
	External calibration in charcoal‐stripped serum	Bile acids	Method for bile acid quantitation in serum	46
	External calibration in de‐lipidized serum	Ceramides / dihydroceramides	Investigate Ceramides in serum during pregnancy	48
	Calibration curve in charcoal depleted cell extracts	Methionine related metabolites	Quantify methionine metabolites in cancer cells	47
	External calibration in solution with IS normalization	Methionine cycle metabolites	Method for methionine metabolites in plasma	33
	External calibration in solution with IS normalization	Metabolites from various classes	Large‐scale method for 560 metabolites in plasma	34
	Standard addition with IS normalization	Amino acids	Method for quantitation of amino acids in serum	7
***In vivo* stable isotope labelled internal standards**	External calibration using ^13^C labelled yeast as IS	Amino acids	Quantitation method for amino acids in plasma	68
	External calibration using ^13^C labelled yeast as IS	CoA and acyl‐CoA thioesters	Quantitation method for short CoA esters	66
	External calibration using ^13^C labelled spirulina as IS	Metabolites from various classes	Quantify key metabolites at different conditions in bacteria	64
	Internal calibration with ^12^C standards	Metabolites from various classes	Quantify ^13^C bacterial metabolites using unlabelled standards	62
	Internal calibration with credentialized ^13^C extract	Metabolites from various classes	Quantifying metabolites in mouse models of cancer	63
**Chemical isotope labeling derivatization**	Girard's reagent T (GT) as chemical reagent	SCFA	Quantify and monitor levels of gut microbial SCFA	89
	DIPP‐Ala‐NHS as chemical reagent	Amino acids	Quantitative profiling of amine metabolites in urine	85
	Dansyl‐Cl (Dns‐Cl) as chemical reagent	Polyphenols	Quantitation method for polyphenols in plasma	94
	DMBA and GT as reagents	Carbonyl / hydroxyl steroids	Quantitation method for steroids in urine	88
	MOBHA derivatization	Monosaccharides	Method for monosaccharides levels in fecal samples	95
	Dansyl hydrazine (Dns‐Hz) derivatization	Fatty acids	Monitor fatty acids in plasma as biomarkers for ESCC	91
	Dns‐Cl, Dns‐Hz, p‐DMAP bromide as reagents	Amine/phenol/carboxyl/carbonyl	Quantitation of metabolites in high‐salt fermented food	105
	Dns‐Cl as chemical reagent	Amino acids / phenol	Quantitation method for asthma/COPD biomarker in urine	86
**Quantitative MS imaging**	MALDI‐qMSI	Tryptophan / kynurenine	Monitor levels of Trp and Kyn in tumor tissue	135
	MALDI‐qMSI	Lactate / glutamate	Quantify amino acids in mouse tumors	136
	IR‐MALDESI‐qMSI	Glutathione (GSH)	Monitor levels of GSH in ovarian tissue	149
	Nano‐DESI‐qMSI	Metabolites from various classes	Method for quantitation of metabolites in single cells	139

DIPP‐Ala‐NHS: diisopropyl phosphoryl alanine *N*‐hydroxysuccinimide, NHS: DMBA: 4‐dimethylaminobenzylamine, MOBHA: O‐(4‐Methoxybenzyl)hydroxylamine, DMAP: 4‐(Dimethylamino)pyridine, IR‐MALDESI: infrared‐matrix assisted laser desorption electrospray ionization, ESCC: esophageal squamous cell carcinoma, COPD: Chronic obstructive pulmonary disease

Further developments would facilitate quantitation in complex samples and enable more detailed studies. The approaches presented in this review, although widely used, still can be improved. One area needing further investigation is the development of artificial matrices for more complex samples. Another important area under development is accessing biosynthesized isotopically labelled metabolites in complex organisms. Having access to well‐characterized and credentialized complex matrices is valuable in metabolomics as it enables more metabolite coverage in quantitative studies involving higher organisms. More comparative studies of the various approaches would also provide a better understanding of the advantages and limitations of each.

The development and increased accessibility of novel technologies will continue to yield breakthroughs in quantitative metabolomics studies. Techniques such as ion mobility or newly adapted ionization methods have become available, enabling new approaches like quantitative single‐cell analysis^143^, ^144^, ^145^ or quantitative metabolic flux analysis.^146^, ^147^ For instance, ion mobility separation can help compensate for the lack of chromatographic separation in mass spectrometry imaging.^148^, ^149^ Quantitative single‐cell metabolomics poses numerous challenges; it is much more difficult to quantify compounds in a single cell than in a larger biological sample. The continual improvement of quantitative analytical techniques, which are increasingly precise and sensitive, will make the analysis of single‐cell metabolomics more attainable. This area of research offers the ability to address the heterogeneity of a sample at the cellular level.

As mentioned above, absolute quantitation of the metabolome is still a crucial challenge. Measuring the absolute concentrations of metabolites in biological samples facilitates comparisons between laboratories or different studies. In the pursuit of this goal, quantitative metabolite databases are emerging. These databases provide access to identified metabolites within a given biological matrix, along with their concentrations as determined in each study. Contributing to the development of such databases will undoubtedly help streamline comparisons in quantitative studies.

## CONFLICT OF INTEREST STATEMENT

The authors declare no conflict of interest.
